# Generation of high oleic acid sunflower lines using gamma radiation mutagenesis and high-throughput fatty acid profiling

**DOI:** 10.3389/fpls.2023.1138603

**Published:** 2023-11-13

**Authors:** Wilfried Rozhon, Veronica E. Ramirez, Silke Wieckhorst, Volker Hahn, Brigitte Poppenberger

**Affiliations:** ^1^ Biotechnology of Horticultural Crops, TUM School of Life Sciences, Technical University of Munich, Freising, Germany; ^2^ KWS SAAT SE & Co. KGaA, Einbeck, Germany; ^3^ Landessaatzuchtanstalt, University of Hohenheim, Willstätt, Germany

**Keywords:** *Helianthus annuus*, HO trait, HPLC, mutagenesis, oleic acid, oil seed crop, sunflower, vegetable oil

## Abstract

Sunflower (*Helianthus annuus L.*) is the second most important oil seed crop in Europe. The seeds are used as confection seeds and, more importantly, to generate an edible vegetable oil, which in normal varieties is rich in the polyunsaturated fatty acid linoleic acid. Linoleic acid is biosynthesized from oleic acid through activity of the oleate desaturase FATTY ACID DESATURASE 2 (FAD2), which in seeds is encoded by *FAD2-1*, a gene that’s present in single copy in sunflowers. Defective *FAD2-1* expression enriches oleic acid, yielding the high oleic (HO) acid trait, which is of great interest in oil seed crops, since HO oil bears benefits for both food and non-food applications. Chemical mutagenesis has previously been used to generate sunflower mutants with reduced *FAD2-1* expression and here it was aimed to produce further genetic material in which FAD2-1 activity is lost and the HO trait is stably expressed. For this purpose, a sunflower mutant population was created using gamma irradiation and screened for *fad2-1* mutants with a newly developed HPLC-based fatty-acid profiling system that’s suitable for high-throughput analyses. With this approach *fad2-1* knock-out mutants could be isolated, which stably hyper-accumulate oleic acid in concentrations of 85-90% of the total fatty acid pool. The genetic nature of these new sunflower lines was characterized and will facilitate marker development, for the rapid introgression of the trait into elite sunflower breeding material.

## Introduction

The sunflower (*Helianthus annuus L.*) is one of our most important oil crops, currently ranking 2^nd^ in Europe and 4^th^ on a global scale (Pilorgé, 2020; Zhou et al., 2020). It holds promise for stable performance under climate change, since it forms long tap roots that reach deep into the soil and confers a substantial degree of drought tolerance (Comas et al., 2013).

Sunflower is a tall, annual plant, with a decorative flower head composed of disc florets in its center and ray flowers that surround it. This is a typical feature of the plant family Asteraceae to which sunflower belongs. Each disc floret develops into an achaene-like fruit called cypsela, which forms one seed covered by a thin pericarp and a cellulose-rich seed coat, termed hull (Seiler, 1997). Botanically the fruit is a nut, and since it synthesizes large amounts of fatty acids, it is used to generate a vegetable oil by press extraction (Dorrell and Vick, 1997).

Standard sunflower oil is rich in the polyunsaturated fatty acid linoleic acid (C18: 2) and is a popular edible plant oil. In addition, it has potential for use in the chemical-technical industry as a substitute for petroleum-based products, if the content of the monounsaturated fatty acid oleic acid (C18: 1) is high (Pilorgé, 2020; Zhou et al., 2020). High oleic acid (HO) oils with an oleic acid content of >85% have high oxidation and heat stability. This increases the shelf life of vegetable oils and prevents the formation of trans-fats when they are heated to high temperatures (e.g. during frying), which is why HO oils are high-quality cooking oils (Zhou et al., 2020).

**Figure 1:**
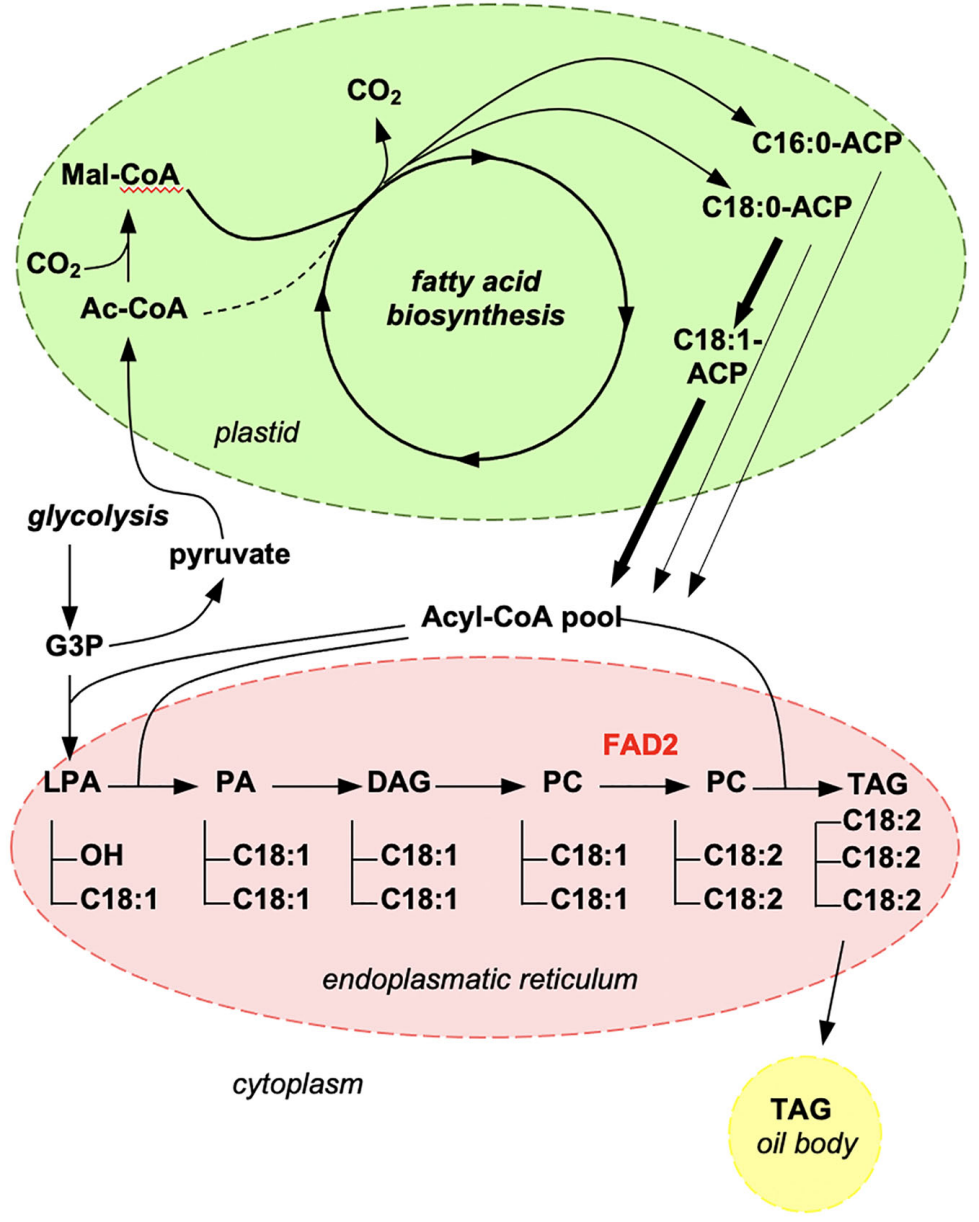
Illustration of key steps of the fatty acid biosynthetic pathway. Oleic acid (C18:1) is synthesized in plastids (in green) and then transported to the endoplasmic reticulum (in red) where it is converted to linoleic acid (C18:2) by FAD2. The generated triacylglycerol (TAG) is stored in oil bodies. AcCoA, acetyl-coenzyme A; ACP, acyl-carrier-protein; DAG, diacylglycerol; G3P, 3-phosphoglycerate; LPA, lysophosphatidic acid; Mal-CoA, malonyl-coenzyme A; PA, phosphatidic acid; PC, phosphatidylcholine; TAG, triacylglycerol.

Seeds of standard sunflower cultivars show an oleic acid content of <30% (Zhou et al., 2020) and thus, a central goal of sunflower breeding is to create new varieties that are rich in oleic acid. Oleic acid is biosynthesized from stearic acid (C18:0) and converted into linoleic acid by the activity of the enzyme oleate desaturase FATTY ACID DESATURASE 2 (FAD2), which is active in the endoplasmic reticulum (**Figure 1**). The FAD2 isoform *FAD2-1* is expressed in seeds (Dar et al., 2017) and if its expression is impaired oleic acid accumulates.

Plants defective in *FAD2-1* expression have been generated with different methods. These included classical plant breeding approaches such as mutation breeding and genetic engineering techniques such as RNAi and Crispr/Cas9, which yielded HO varieties of rice, soybean and rapeseed (Pham et al., 2012; Abe et al., 2018; Long et al., 2018).

In sunflower most currently available HO varieties are based on the Pervenets line, which was created through chemical mutagenesis (Soldatov, 1976), yielding a duplication of the promoter region of *FAD2-1*. This induces silencing of *FAD2-1* and thereby reduced FAD2-1 expression and activity (Schuppert et al., 2006). However, since the *FAD2-1* promoter is still active and highly regulated by environmental cues (Baldini et al., 2014), oleic acid concentrations in Pervenets-based varieties strongly fluctuate, depending on the growth conditions (Miller et al., 1987; Fernández-Martìnez et al., 1989; Grunvald et al., 2013; Van Der Merwe et al., 2013). In addition, a *fad2-1* loss-of-function mutant was produced by chemical mutagenesis, which contains a large insertion in the *FAD2-1* coding sequence and stably hyper-accumulates oleic acid (Alberio et al., 2016; Alberio et al., 2018).

Here we aimed to generate additional *fad2-1* knock-out mutants of sunflower, to create new genetic resources for breeding progress in this area. For this purpose, a high-throughput, HPLC-based screening system was developed and used to screen a sunflower mutant population that was generated with gamma irradiation. HO sunflower mutants were isolated and genetically characterized, which identified individuals with single point mutations in *FAD2-1* that result in a stable hyper-accumulation of oleic acid, at concentrations of up-to >90%. The implications of these new genetic resources for progress in breeding elite HO sunflowers cultivars are discussed.

## Materials and methods

### Mutagenesis of sunflower seeds

For the generation of a mutagenized sunflower population, 2 kg seeds of the American restorer line RHA857 were exposed to a Cobalt 60 gamma source with 300 Gy at the Department of Nuclear Sciences and Applications of the International Atomic Energy Agency in Vienna, Austria. In spring 2017 20,000 seeds were sown in a field in Willstaett, Germany, where they showed an adequate germination rate and were grown to the adult stage. 2,300 randomly selected plants were selfed and harvested. Additionally, 3,660 open-pollinated plants were harvested. One seed per harvested plant was sown in 2018 in the field to generate M2 plants, which all grew in summer 2018 in Willstaett, Germany, together with the parent line in normal weather conditions. All plants were bagged for selfing and harvested individually. The seeds of these M2 plants were then screened.

### Development of a HPLC-based methodology for fatty acid quantification from seeds

The fatty acid detection protocol was optimized from previously published hydrolysis-based approaches, which had used EtOH in combination with KOH (Eremina et al., 2015). Due to a rapid yellowing of the hydrolysis reagent, a mixture of 3 M KOH and 1-propanol, which was stable for least 6 months at 4°C, was used instead.

The HPLC LC-10 system (Shimadzu, Kyoto, Japan) consisted of a DGU-14A degasser, a LC-10AT pump with a low-pressure gradient valve, a SIL-10A auto-sampler, a CTO-10AS column oven, a SPD-10A UV detector that was operated at 260 nm and a SCL-10 system controller. Chromatograms were recorded and analyzed with Clarity™ software package (DataApex, Prague, Czech Republic). Separation was performed using a Nucleodur C8 Gravity 1.8 µM 50x3.0 mm column (Macherey-Nagel, Düren, Germany), which was maintained at a temperature of 40°C. Acetonitrile/methanol/water=50/36/14 (v/v/v) was used as an eluent and applied at a flow rate of 0.5 ml/min with a pressure of approximately 125 bar.

### Fatty acid profiling of seed pools from M2 plants with HPLC

**Figure 2:**
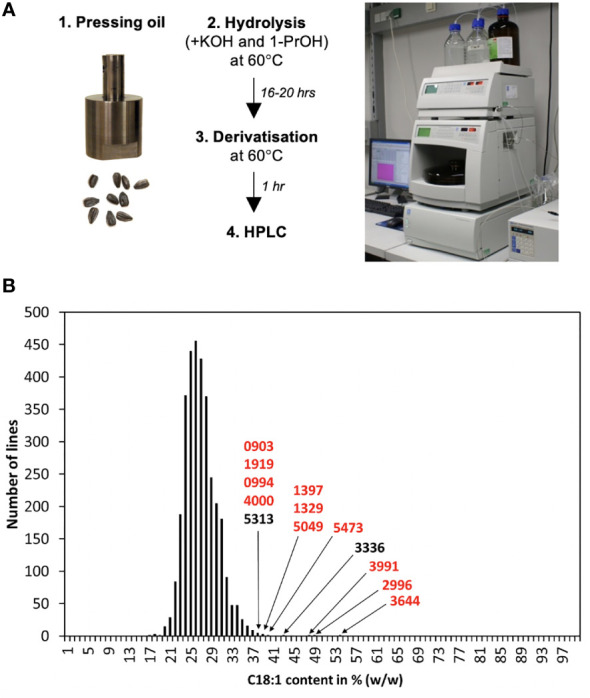
A high throughput fatty acid profiling system and its application to screening a sunflower mutant population. **(A)** Layout of the protocol developed for the screen. Oil obtained by press extraction with a custom-made press was used directly for hydrolysis and derivatization of fatty acid, which were quantified by UV-detection-based HPLC. **(B)** 3,269 mutant lines were analyzed with the protocol and grouped according to their C18:1 content (in % of the total fatty acid pool). 99,5% of these mutant line pools had a C18:1 concentration of 20-38%, but 13 pools showed higher amounts; their identifiers are shown.

Ten to twenty seeds were pooled per line and used to extract about 50-200 µl of oil by press extraction with a custom-made stainless-steel pressing tool (**Figure 2A**; **Supplementary Figure S1**) placed in a P400 hydraulic press (Sirio Dental SRL, Meldola, Italy) that was used to apply a force of about 3 t. A 5 µl aliquot of this oil was transferred to a 1.5 ml safe-lock tube (Eppendorf, Hamburg, Germany), mixed with 500 µl of hydrolysis reagent (6 M KOH/l-propanol = 5/95) and incubated overnight at 60°C. 10 µl of this reaction were then transferred to a ND9 HPLC tube (Macherey-Nagel, Düren, Germany), mixed with 1,000 µl of derivatizing reagent (750 mg 2,4′-dibromoacetophenone and 150 mg 18-crown-6 ether dissolved in 500 ml acetonitrile), sealed with an M1 crimp cap (Macherey Nagel, Düren, Germany), and incubated for 1 hour at 60°C. From this solution 10 µl were injected into the HPLC system.

### Single seed fatty acid measurements

For fatty acid quantification in single seeds, a section of the seed where the cotyledons are located (the round tip) was cut off with a knife, weighed, transferred into a safe-lock 2 ml reaction tube and homogenized with an MM 400 mixer mill (Retsch GmbH, Haan, Germany) using a steal bead with a diameter of 3 mm (GAP Kugellager OHG). Subsequently, for each mg homogenized plant material 50 µl hydrolysis reagent was added and the tubes were incubated at 60°C overnight. On the next day the tubes were centrifuged (13.000 rpm, 5 min) and 10 µl supernatant was derivatized as described above. The rest of the seed was placed for 48 hours in water to induce germination and the seed was transferred to soil once the radicle had emerged.

### Sequencing of isolated lines

For the genetic characterization of isolated HO mutants, DNA was extracted from leaves using the E.Z.N.A.^®^ plant DNA isolation kit D3485 (Omega Bio-tek, Norcross, GA, USA) and used for PCR amplification of the *FAD2-1* locus regions with the primer pairs F2 (5’-GAAAAGTCTGGTCAAACAGTCAACAT-3’), F5 (5’-GTAACGTCTGCGCGCTTGCAGACATCA-3’), R2 (5’-CCGATGTCGGACATGACTATC-3’), R4 (5’-TCAGGTCAAAACGAGCTGTG-3’) and R10 (5’-GACAGCGGTTATGGTGAGGT-3’) (Schuppert et al., 2006). The PCR products were subcloned into the pGEM-T-Easy cloning vector (Promega, Madison, WI, USA) and sequenced by a commercial service (Eurofins Genomics, Ebersberg, Germany).

The obtained sequences were aligned to the *FAD2-1* consensus sequence from the background line RHA857 using Clustal Omega (EMBL-EBI, Hinxton, UK). Single point mutations were identified and coordinates assessed to determine amino acid changes and resulting functional mutations.

## Results

### A high-throughput method for fatty acid quantification in sunflower seeds

The work was initiated with the optimization of a fatty acid quantification procedure that applies UV detection-based HPLC (Eremina et al., 2015). For sample preparation, the reference protocol involved homogenizing 10 mg seed material with liquid nitrogen, a 16-20 h extraction with KOH/EtOH/H_2_O, an acidification with H_3_PO_4_, an extraction with heptane, evaporation of the organic phase to dryness and a derivatization with 2,4′-dibromoacetophenone in the presence of 18-crown-6 ether and potassium carbonate at 60°C for 1 h. This sample preparation method was improved, in particular in terms of hydrolysis and derivatization; more specifically it was tested if the acidification and heptane extraction steps are required for an efficient quantification of the fatty acids, C16:0 (palmitic acid), C18:0, C18:1 and C18:2. The results showed that these steps could be omitted without negative effects on the reproducibility of the system (**Supplementary Figure S2**). Oil obtained from coated seeds had a slightly lower C18:1 and a slightly higher C18:2 content than oil extracted from peeled seeds. However, since these differences were minute, coated seeds were used for the subsequent experiments because that simplified the procedure. Moreover, it was tested and found that oil pressed from the seeds with a custom-made press (**Supplementary Figure S1**) could be directly used for the measurements. This simplified sample preparation and yielded a final set-up that only required hydrolysis and derivatization of the seed oil (**Figure 2A**).

In addition to refining the sample preparation, the eluent composition was optimized by adding methanol, which improved separation of palmitic and oleic acid. Moreover, instead of a LiChrospher 60 RP-select B 5 µm 125x4 mm column, a Nucleodur C8 Gravity 1.8 µM 50x3 mm column was chosen. Both measures together allowed reducing the eluent volume from 15 ml to 5 ml (**Supplementary Figure S3**).

To develop the screening procedure, it was also tested if within a M2 seed pool, single seeds homozygous for *FAD2-1* knock-out mutation that were expected to over-accumulating oleic acid at levels of >80%, would be detectable. For this purpose, pools of wild-type seeds were spiked with individual seeds of the HO cultivar BE302 bearing the Pervenets mutation (Soldatov, 1976) and the fatty acid profile of the whole pool was determined with HPLC. This showed that using a pool of 10 and 20 seeds, a heterozygous line would be detected with a chance of 75% and 91%, respectively (data not shown). Thus, 20 seeds were used for analysis, except for lines where only few seeds were available; for those 10 seeds were used. These estimations are valid under the assumption that wild type and heterozygous seeds both have an oleic acid fraction of 26% and HO seeds of >80%. However, as it turned out later, heterozygous seeds had an oleic acid fraction of approximately 35% and thus the identification rate should be close to 100% using either 10 or 20 seeds.

### A screen of a mutagenized sunflower population identifies HO sunflower mutants

The sunflower population was generated using gamma irradiation of seeds of the American fertility restorer line RHA857. A number of developmental mutants were present among these M2 plants (data not shown), showing that the irradiation had been successful in producing mutant plants. Seeds of all generated M2 lines were harvested, the seed amount was determined and if it was above 10 seeds, the line was included. This left 3,269 lines, which were analyzed with the developed HPLC-based method.

The results of the measurements are summarized in **Figure 2B**. The majority of the samples (3,253 out of the 3,269 = 99,5%) showed oleic acid concentrations of about 20-38% of the total fatty acid pool (**Figure 2B**). However, in 13 lines the oleic acid levels of the analyzed seed pool exceeded 38%; these were considered of interest and selected for further analysis. Among them, lines 2996, 3644 and 3991 showed the highest values with approximately 48-54% oleic acid (**Figure 2B**) and these were analyzed further.

### Non-destructive single seed measurements facilitated the selection of homozygous lines

**Figure 3:**
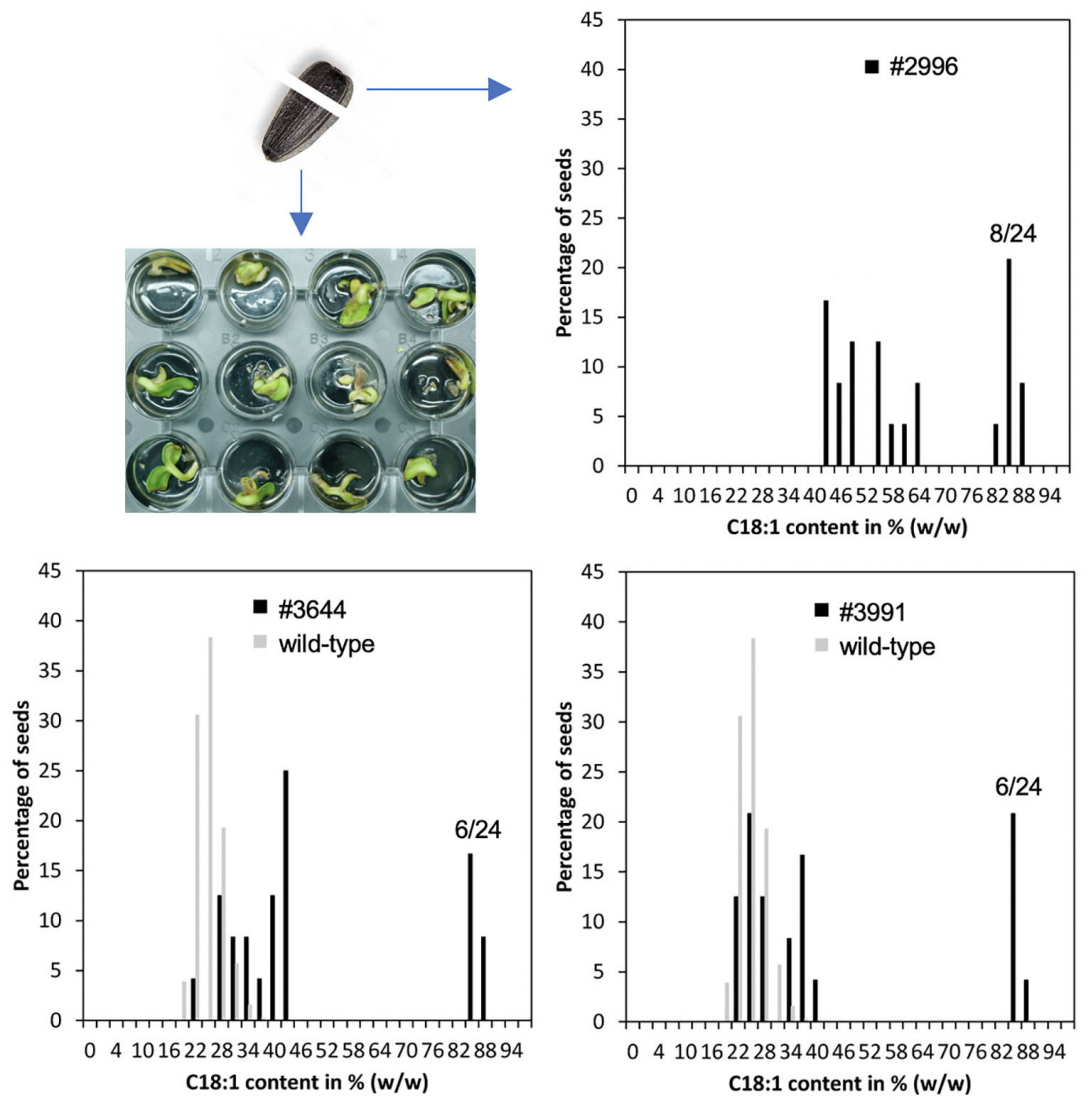
Non-destructive single seed measurements were used to isolate lines homozygous for the HO trait. Left: The round tips of single sunflower seeds were cut off and used for fatty acid analyses. The rest of the seeds were imbibed in water for germination. Right: In M2 seed pools of the HO mutant lines #2996, #3991 and #3644, the HO trait segregated in an almost perfect 3:1 Mendelian fashion with 6-8 out of 24 analyzed seeds showing oleic acid fractions of approximately 86-88% (dark grey). The average levels of the mutagenized population are shown in light grey.

To test, if the seed pools of the identified three lines may contain individuals with increased oleic acid concentrations, 24 single seeds from each M2 pool were analyzed. For this purpose, the round tip of the seed, where the cotyledons are embedded, was cut off with a razor blade, homogenized with a mill and subjected to HPLC analysis. The rest of the seed was submerged in water for germination and transferred to soil, if needed (**Figure 3**).

The results of these single seed measurements showed that approximately 25% of the seeds of lines 2996, 3644 and 3991 had oleic acid concentrations of well above 80% (**Figure 3**), indicating that a single locus that confers a recessive trait was affected. The rest of the seeds had oleic acid concentrations that were either similar to the average of the whole population (22-28%; **Figure 3**) or clearly higher, implying that even in a heterozygous situation, there was a certain penetrance of the HO phenotype (**Figure 3**).

**Figure 4:**
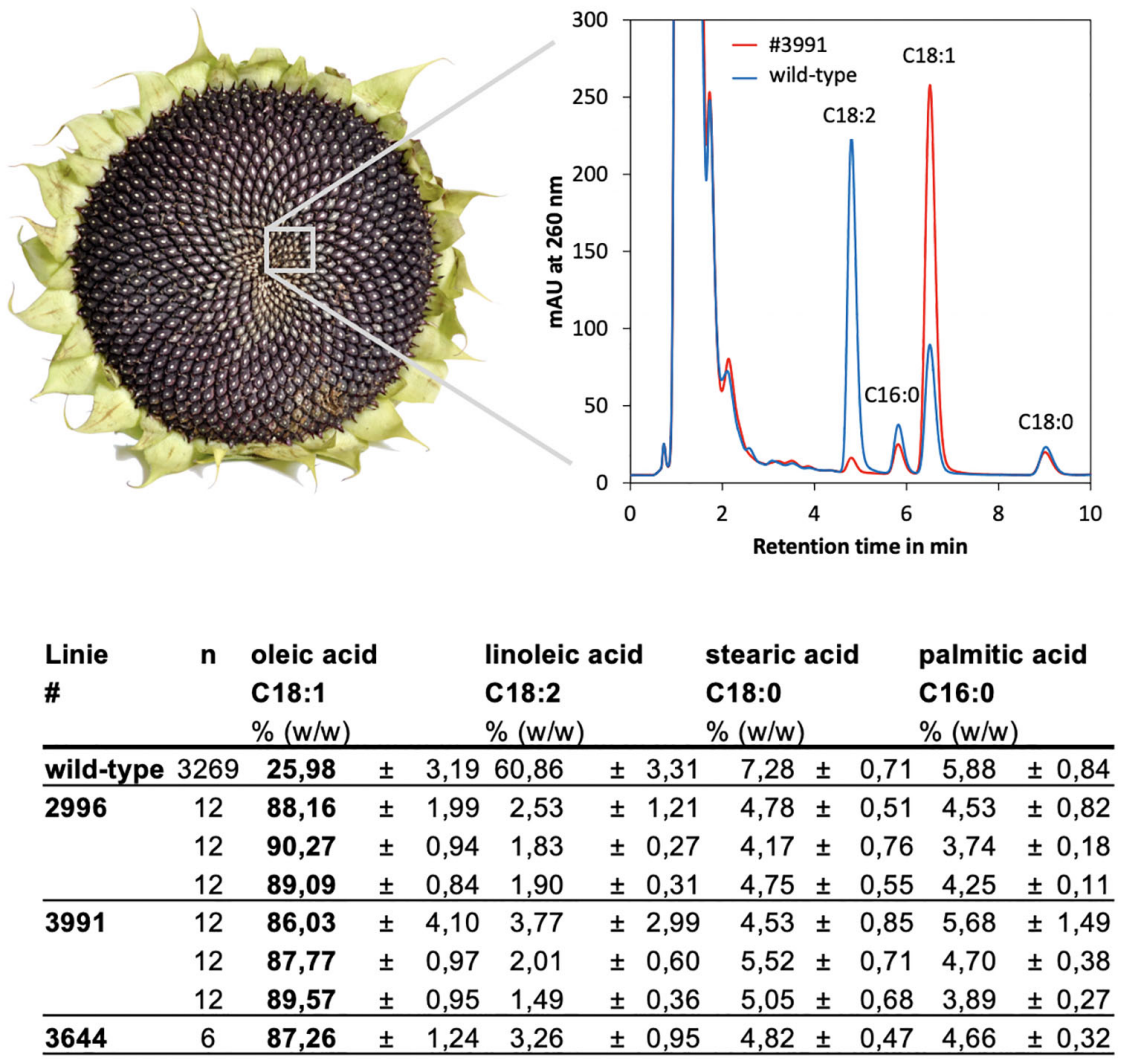
Fatty acids were quantified in single seeds of the M3 generation of the isolated HO mutants by HPLC. Top: The chromatograms showed clear shifts from C18:2 to C18:1 in the HO mutants as compared to the wild-type line RHA857. A strong increase in C18:1 at the expense of C18:2 was obvious. Bottom: Fractions of oleic acid (C18:1), linoleic acid (C18:2), stearic acid (C18:0) and palmitic acid (C16:0) in the average of the whole population as compared to the isolated mutants. For lines #2996 and #3991 the results of three independent progeny lines are shown, of which 12 seeds each (n) were measured. For line #3644 the results of 6 seeds that originated from one independent, homozygous line, are given.

The remaining parts of the individual HO seeds were used directly to amplify the M3 generation of the identified lines. From this M3 generation again single seeds were measured, to analyze if the trait was homozygous and if it was stably inherited. The results showed that the progeny of all lines had oleic acid fractions of 86-90% (**Figure 4**). Linoleic acid was also measured and found to be reduced from about 60% in the parent line (average of the mutant population) to 2-4% in the HO mutants. Stearic and palmitic acid accounted for the remaining 10% of the fatty acid pool and thus were only slightly reduced as compared to the parent background with a level of approximately 13% (**Figure 4**); this could be due to an increased flux through the biosynthetic pathway.

### The isolated HO sunflowers contain single point mutations or deletions in *FAD2-1*

The nature of the HO chemotype and its recessive, monogenetic inheritance indicated that *FAD2-1* expression was defective in the three isolated lines. To test if this may be caused by mutations in the gene or its up-stream or down-stream untranslated regions (UTRs), the locus was sequenced. The results showed that lines #2996 and #3991 contained single nucleotide deletions in the *FAD2-1* coding sequence, which induced reading frame shifts that yielded non-sense amino acid sequences and premature stops at amino acid 331 (**Supplementary Figure S4**). More specifically, in line #2996 T944 of *FAD2-1* was deleted resulting in the mutant protein FAD2-1^L315C.fs^. In #3991 T918 of *FAD2-1* was deleted resulting in the mutant protein FAD2-1^T307P.fs^. Line #3644 contained a very large deletion, where the whole *FAD2-1* locus, including its 5’ and 3’ UTR, was deleted. Thus, in all three lines the *FAD2-1* locus was mutated, which correlated with oleic acid levels of up to 90%.

## Discussion

Oil quality is an important trait in oil crops and generating cultivars with high oleic acid levels has been a major aim of breeding in the past years. HO lines of several species have been developed by employing mutagenesis or silencing of *FAD2-1*. In soybean, which possesses two functional *FAD2-1* copies, conventional mutation breeding was used to generate a *fad2-1axfad2-1b* double mutant, which accumulates oleic acid to approximately 80% (Pham et al., 2012). In rapeseed, the super-high oleic acid line N1379T was created by combining parents, each mutated in one of the two functional *BnFAD2* versions, to generate a *fad2-1-Axfad2-1B* double mutant, which has oleic acid concentrations of 85% (Long et al., 2018).

Sunflower has only one *FAD2-1* copy and chemical mutagenesis was used in the past, to generate HO sunflower lines that are defective in *FAD2-1* expression. The most important material initially produced in this way was the Pervenets line, which has since been used in breeding programs (Soldatov, 1976). However, as it is based on a duplication of the promoter region, the HO trait is unstable. Moreover, since no single-nucleotide polymorphisms in the *FAD2-1* region are present (Schuppert et al., 2006), inexpensive, diagnostic markers that would facilitate breeding can’t be developed. In addition to Pervenets, a sunflower *fad2-1* mutant called NM1 has been described, which contains a large insertion in *FAD2-1* (Alberio et al., 2016; Alberio et al., 2018). The same authors generated additional mutants with base changes in *FAD2-1*, which were patented (León et al., 2013a; León et al., 2013b).

To generate new genetic resources with abolished *FAD2-1* expression, a sunflower mutant population was generated with gamma irradiation and screened with an HPLC-based fatty acid profiling system specifically developed for the screen. The method was suitable even for a non-destructive approach, the measurements of seed parts, which made a rapid selection of lines, homozygous for the HO trait-conferring mutations possible. In these lines the *FAD2-1* locus region was sequenced and it was found that one contained a large deletion and two contained single nucleotide deletions, which induced frame shifts and amino acid changes in the C-terminal region of FAD2-1, that can be expected to yield abolished FAD2-1 activity (Zambelli and León, 2015. The frame shifts led to non-sense amino acid sequences starting from aa position 315 in line #2996 and starting from aa position 307 in line #3991, yielding FAD2-1 ^L315C.fs^ and a FAD2-1 ^T307P.fs^ proteins and premature stops (**Supplementary Figure S4**). For these deletions it is possible to develop cost-effective assays that specifically detect the deletions and can be used for marker-assisted selection. Thereby, homozygous and heterozygous carriers as well as non-carriers can be distinguished and selected.

In the sunflower lines developed here, oleic acid accumulates to levels of 85-90% and this was found to be stable across generations, which is clear evidence that the mutant FAD2-1 versions are inactive. It can therefore be assumed that neither additional genetic factors, such as modifier loci in other genetic backgrounds, nor environmental cues will impact the oleic acid contents. This will enable a rapid conversion of non-HO elite cultivars, by introducing the modified *FAD2-1* gene using marker-assisted selection. Moreover, it will enable the production of HO sunflowers independent of environmental conditions, a clear advantage for the marketing and production of such new cultivars.

## Data availability statement

The original contributions presented in the study are included in the article/**Supplementary Materials**. Further inquiries can be directed to the corresponding author.

## Author contributions

Planned and designed research: all authors. Performed research: WR, VR and VH. Analyzed data: WR, VR, VH and BP. Wrote the manuscript: BP. All authors contributed to the article and approved the submitted version.
