# Supplementary material for: Generation of high oleic acid sunflower lines using gamma radiation mutagenesis and high-throughput fatty acid profiling
Source: Front Plant Sci. 2023 Nov 13;14:1138603. doi: 10.3389/fpls.2023.1138603 (PMC10679672; doi:10.3389/fpls.2023.1138603)
Supplement: Supplementary file 1 [file DataSheet_1.pdf]

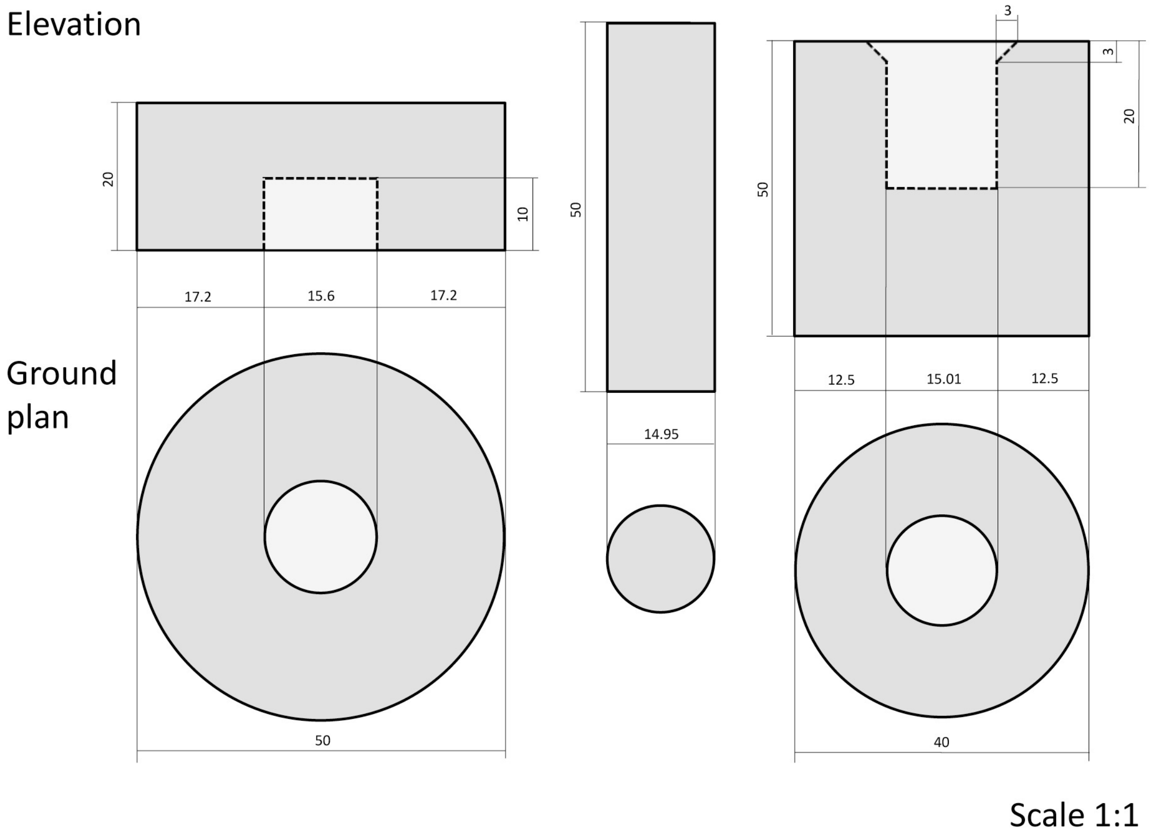


**Supplementary Figure S1.** Sketch of the press extraction tool. The dimensions are given in mm. The extraction tool was made from stainless steel. It can be used to press 10 to 20 sunflower seeds with the help of a hydraulic press that applies a pressure of 3 t.

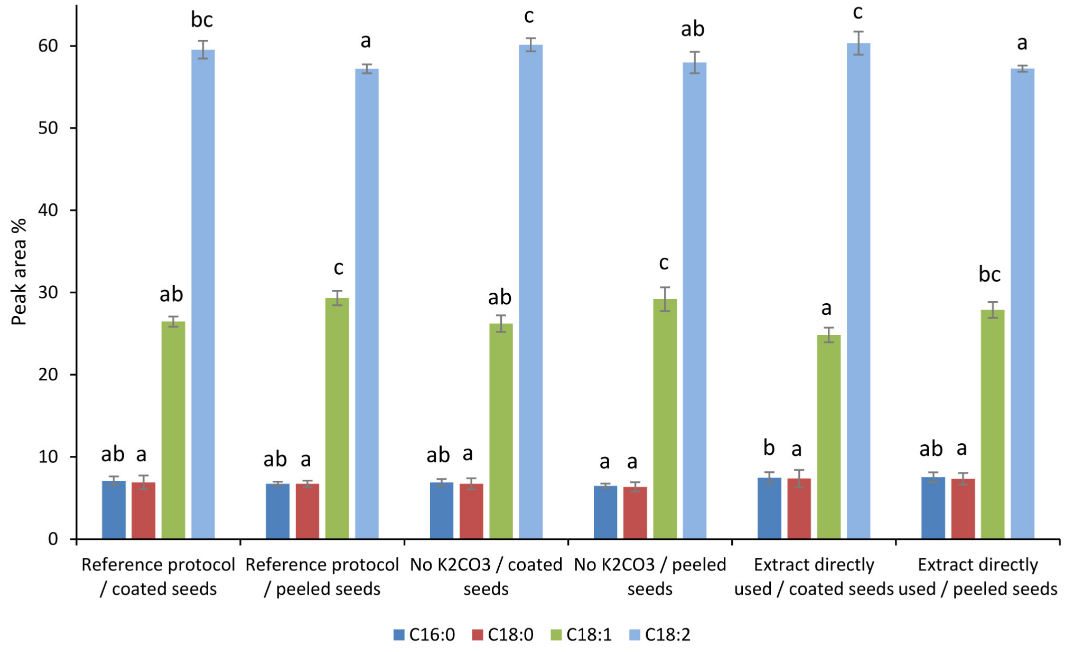


**Supplementary Figure S2.** Results of fatty acid quantification using different extraction procedures. Statistically significant differences at $P < 0.05$ are indicated with different letters and were determined with one-way ANOVA with a post-hoc Tukey HSD test.

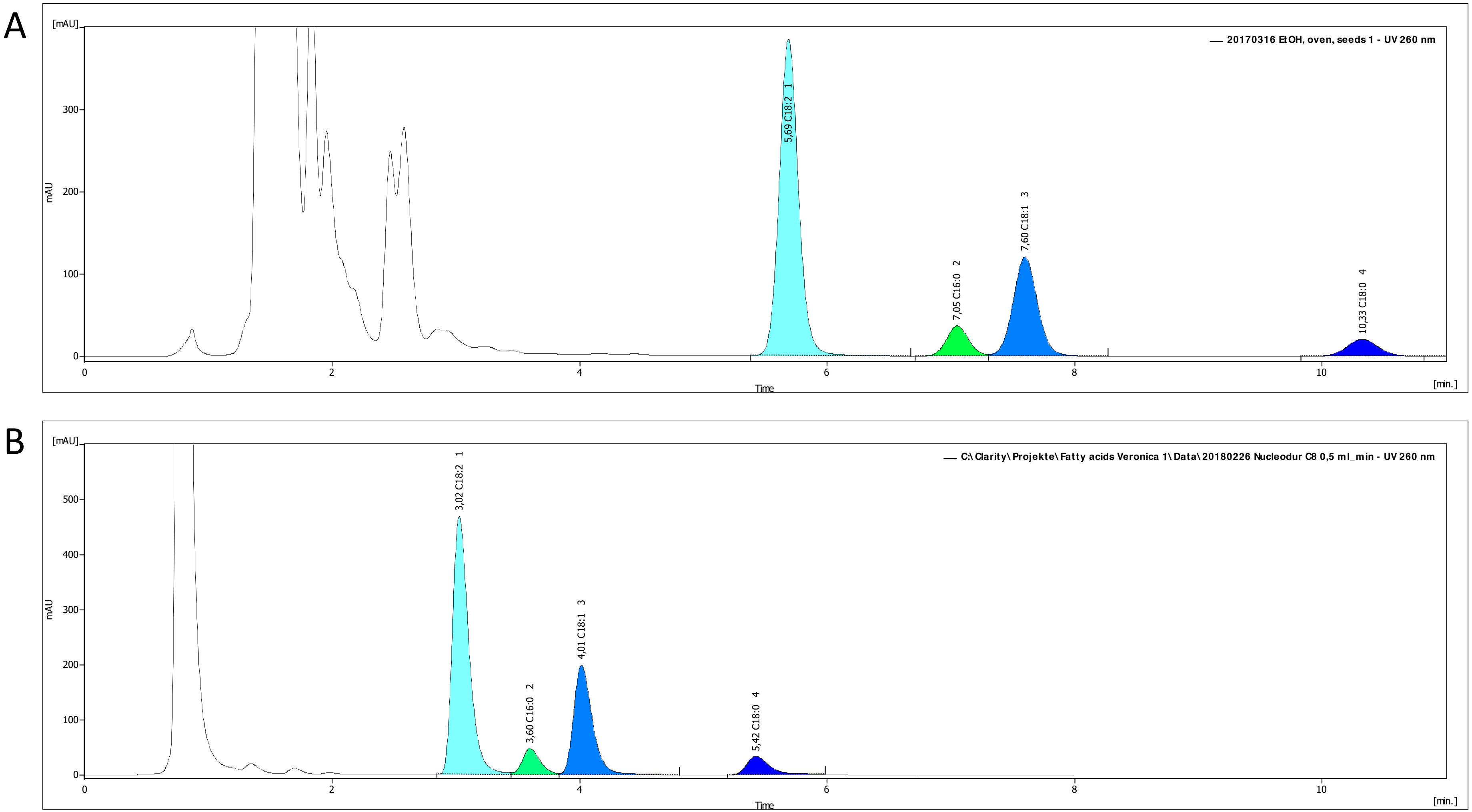


**Supplementary Figure S3.** Optimization of the HPLC conditions. **A**. Separation of derivatized fatty acids of sunflower oil on a LiChrospher 60 Select B 5 µm 125x4 mm column using ACN/$H_2O$=86/14 (v/v) as eluent at a flow rate of 1 ml/min and a oven temperature of 25°C. A total time of 11 min and 11 ml eluent are required. **B**. Separation of the same sample on a Nucleodur C8 Gravity, 1.8 µm 50x3 mm column using ACN/MeOH/$H_2O$=50/36/14 as eluent at a flow rate of 0.5 ml/min and an oven temperature of 40°C. Only 6 min and a volume of 3 ml eluent are required for one sample.

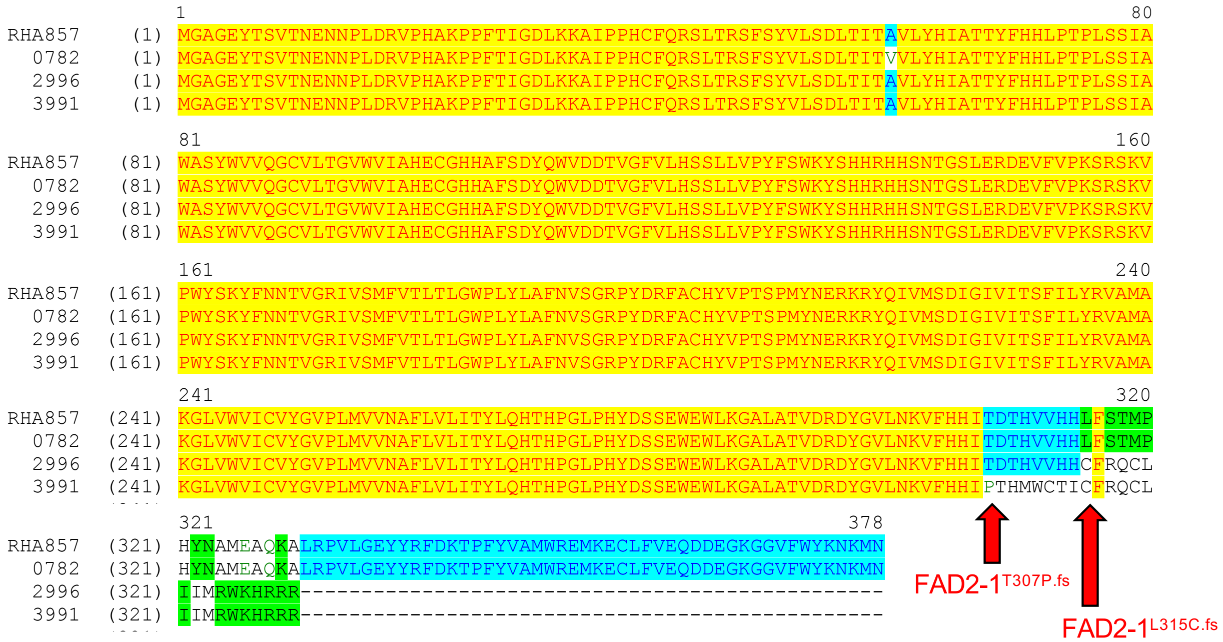


**Supplementary Figure S4.** Amino acid (aa) sequence alignment of the FAD2-1 sequence of the parent line RHA857 and an unrelated line (#0782), with the mutant lines #2996 and #3991, where single nucleotide deletions had occurred. These deletions led to frame shifts and non sense amino acid sequences, starting with the replacement of a proline (P) with a threonine (T) in line #2996 and a replacement of a leucine (L) with a cysteine (C) in line #3991. In both FAD2-1 mutant proteins a premature STOP at aa 331 then occurred. Yellow: identical aa in all lines; Blue: identical aa in three lines; Green: identical aa in two lines.
